# Gastroesophageal Reflux Disease and Tooth Erosion

**DOI:** 10.1155/2012/479850

**Published:** 2011-12-12

**Authors:** Sarbin Ranjitkar, John A. Kaidonis, Roger J. Smales

**Affiliations:** School of Dentistry, Faculty of Health Sciences, The University of Adelaide, North Terrace, Adelaide, SA 5005, Australia

## Abstract

The increasing prevalence of gastroesophageal reflux disease (GERD) in children and adults, and of “silent refluxers” in particular, increases the responsibility of dentists to be alert to this potentially severe condition when observing unexplained instances of tooth erosion. Although gastroesophageal reflux is a normal physiologic occurrence, excessive gastric and duodenal regurgitation combined with a decrease in normal protective mechanisms, including an adequate production of saliva, may result in many esophageal and extraesophageal adverse conditions. Sleep-related GERD is particularly insidious as the supine position enhances the proximal migration of gastric contents, and normal saliva production is much reduced. Gastric acid will displace saliva easily from tooth surfaces, and proteolytic pepsin will remove protective dental pellicle. Though increasing evidence of associations between GERD and tooth erosion has been shown in both animal and human studies, relatively few clinical studies have been carried out under controlled trial conditions. Suspicion of an endogenous source of acid being associated with observed tooth erosion requires medical referral and management of the patient as the primary method for its prevention and control.

## 1. Introduction

Both endogenous (intrinsic) acid and exogenous (extrinsic) sources of acids are responsible for the increasing incidence and high prevalence of tooth erosion and associated tooth sensitivity observed in many countries, in both children and adults [1]. Not only may the tooth erosion from endogenous acid be more severe than that from exogenous acids but also gastric reflux, regurgitation, and microaspiration may have significant adverse effects on the mucosa of the esophagus, oropharynx, and respiratory system [2–6].

A recent systematic review found a median prevalence of 24% for tooth erosion in patients with gastroesophageal reflux disease (GERD) and a median prevalence of 32.5% for GERD in adult patients who had tooth erosion [7]. Therefore, from their observations of tooth erosion, dentists may be the first persons to diagnose the possibility of GERD, particularly in the case of “silent refluxers.” This diagnosis is important, as GERD has increased in prevalence in many countries, and may have severe health effects if not adequately treated [8, 9]. Consequently, dentists should be more aware of the various manifestations of GERD in both children and adults.

## 2. Gastroesophageal Reflux and Antireflux Barriers

Gastroesophageal reflux (GER) is defined as a normal, physiologic retrograde flow of gastric contents into the esophagus that occurs mostly postprandial (after meals) for around one hour per day [10]. A GER episode is diagnosed when esophageal pH drops below 4.0 for at least 30 seconds [11]. But, in healthy individuals, the acidic reflux is cleared by esophageal peristalsis and saliva within 1-2 minutes [12]. Saliva also helps to buffer (neutralize) esophageal acid [13] and to lubricate the esophagus against mechanical damage from a food bolus [10].

An antireflux barrier at the gastroesophageal junction is formed by normal anatomical features, including the oblique course of the gastroesophageal junction and diaphragmatic curve. Of particular importance is a high-pressure gradient of 10–30 mm Hg maintained by tonic contraction of the circular muscles of the lower esophageal sphincter (LES). Luminal clearance of the esophagus is aided by gravity when upright, by physiological emptying (peristalsis) of the esophageal contents into the stomach and by salivary bicarbonate [14, 15].

## 3. Pathophysiology of Gastroesophageal Reflux Disease

GER does not produce gastric symptoms or mucosal damage, but can progress into a clinical disorder termed gastroesophageal reflux disease (GERD), usually characterized by symptoms of heartburn and acid regurgitation [16]. A global definition and classification of GERD has been developed by the Montreal consensus group, based on voting by 44 physicians from 18 countries on several evidence-based statements on the characteristics of GERD. [17]. GERD has been defined as “a condition that develops when the reflux of stomach contents causes troublesome symptoms and/or complications,” and its manifestations have been subclassified into esophageal and extraesophageal syndromes [17]. Recently, attempts have been made to define GERD specifically for the pediatric population (including infants, children, and adolescents) in light of observations of a wider range of variability in the signs and symptoms in children compared with adults [18].

**Figure 1:**
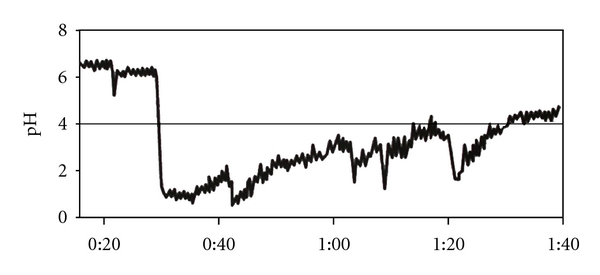
Pattern of sleep-related esophageal acid exposure in a patient with erosive esophagitis. Night-time acid reflux during supine sleep leads to pH levels <4 that are continuous and sustained. (Reproduced Figure 2, page 111S, from Orr [26]. With copyright permission from Elsevier).

GERD can occur both during sleep (nocturnal) and waking (day time) stages, and 40–81% of individuals who reported symptoms of GERD also experienced symptoms during sleep [19–23]. Sleep-related GERD occurs more frequently during the lighter nonrapid eye movement (non-REM) sleep, particularly during the first two hours of sleep, than during the REM stage [12, 24]. These episodes also occur less frequently, but last for longer periods, than those during the waking stage [25]. Each sleep-related GERD episode has been noted to typically last for 15–20 minutes compared with 1-2 minutes during the waking stage [12]. These episodes can recur continuously to lower the esophageal pH below 4.0 for a period of around 60 minutes, including a period of 10 minutes when esophageal pH stays at 1.0, until the pH gradually recovers to above 4.0 (Figure 1) [26]. This situation demonstrates the potential for erosive damage to both the esophageal and extraesophageal structures during sleep-related GERD.

The body has several mechanisms to protect the esophagus against the effects of acid reflux during the waking period. Acid contact on the mucosa in the distal esophagus (near the gastroesophageal junction) during the waking state induces increased salivary flow and swallowing mechanisms (primary peristalsis), and localized esophageal peristalsis (secondary peristalsis) to buffer the acid and facilitate volumetric clearance [12, 27]. These antireflux protective mechanisms also occur during sleep, but at a diminished level. Salivary flow is greatly reduced during sleep, but saliva secreted intermittently in response to orofacial movements, such as chewing-like jaw activity or rhythmic masticatory muscle activity, helps to lubricate the esophageal mucosa [28]. An experimental study on healthy individuals has shown that acid infusion into the esophagus during sleep resulted in a brief period of arousal with a swallowing reflex, which was also believed to promote saliva flow [29]. Therefore, sleep-related acid reflux may induce similar responses, as well as the perception of heartburn, which are known to be important mechanisms responsible for salivary secretion, volumetric clearance of the refluxate, and the prevention of pulmonary aspiration [30]. Even though these antireflux mechanisms operate in the majority of GERD patients during sleep, there is still a greater risk of proximal migration of refluxate as well as an increased duration of acid-mucosa contact during sleep compared with the waking state [12, 31]. Thus, sleep-related GERD poses a greater risk of developing esophageal complications (including reflux esophagitis) and extraesophageal complications (including respiratory tract conditions and pulmonary microaspiration of the refluxate) compared with the waking state [23, 26, 31]. In essence, GERD disturbs sleep and sleep disturbances worsen GERD [32, 33].

## 4. Risk Factors for GERD

GERD is usually caused by a transient relaxation of the LES and less commonly by a transient increase in intra-abdominal pressure or a low resting pressure of the LES [34]. Generally recognized risk factors for gastric regurgitation include conditions that cause LES incompetence (alcohol, nicotine, caffeine, many medications, and hiatal hernia), conditions that cause increased intra-abdominal pressure (obesity, pregnancy, straining, and bending), and conditions that cause increased gastric volume (heavy meals and intestinal obstruction). Alcohol consumption may also increase gastric acid secretion and delay gastric emptying, and nonsteroidal anti-inflammatory drugs may interfere with prostaglandin cytoprotection [35]. Obstructive sleep apnoea (OSA) and obesity predispose to nocturnal GERD, with more than 100 reflux episodes reported during an 8-hour sleep in individuals suffering from OSA [36, 37]. The consumption of spicy and acidic foods and beverages may also aggravate GERD problems.

## 5. Diagnosis of GERD

Common methods for the diagnosis of GERD include the assessment of gastric symptoms, a proton pump inhibitor (PPI) drug test, esophageal pH monitoring, and upper endoscopy [8].

As both gastric and duodenal reflux occur frequently in individuals suffering from GERD, a combined assessment is important in obtaining a holistic understanding of its pathophysiology [38]. Esophageal symptoms can be associated with either acid or bile or a combination of both in GERD patients, but the majority of symptoms are associated with gastric acid [39]. Furthermore, duodenal refluxate in the absence of gastric refluxate does not cause reflux esophagitis [40].

Classical symptoms of GERD in adults are heartburn and acid regurgitation causing a sour taste, with less common symptoms being dysphagia (difficulty swallowing), water brash (flooding of the mouth with saliva), odynophagia (pain on swallowing), burping/belching, chronic cough/hoarse voice, nausea, and vomiting [41]. However, cultural differences and language barriers need to be considered in diagnosing GERD because of the difficulties associated with direct translation of English words (such as heartburn) into other languages [8]. Also, it is obvious that an assessment of symptoms alone will be unable to detect instances of “silent GERD.”

It is generally agreed that the overall management of GERD should focus on reducing acid regurgitation with the use of PPIs initially, and antireflux surgery if required subsequently [40]. In the absence of serious symptoms and signs, PPIs administered over 1–4 weeks are a cost-effective initial treatment therapy and diagnostic test for GERD [42]. If regurgitation symptoms fail to respond to this treatment, patients are usually followed up with pH-monitoring studies [8, 43].

pH monitoring is considered to have the highest sensitivity (ability to detect true cases as positive) and specificity (ability to diagnose false cases as negative) in diagnosing GERD [44]. pH-monitoring systems include a 24-hour catheter-based pH-monitoring system and a 48-hour wireless pH-monitoring system. The latter system causes less interference in daily life activities and has higher sensitivity and specificity than the former [43, 45, 46].

Assessment of symptoms and pH monitoring are not reliable for detecting erosive changes in the esophageal mucosa. Reflux esophagitis, referring to the injury with inflammation of the esophagus from gastric refluxate, is a common manifestation of GERD that is recognized during endoscopy [16]. However, in one study, most patients showed only mild or no erosion of the esophageal mucosa [47]. Endoscopy is also used to detect Barrett's esophagus and hiatal hernia and for sampling for the presence of *Helicobacter pylori* from gastric mucosa [46, 48].

## 6. Advanced Esophageal Manifestations of GERD

Severe forms of GERD have been associated with Barrett's esophagus, which is a form of esophageal metaplasia characterized by aneuploidy (abnormal number of chromosomes) [2]. This condition can progress to low-grade and high-grade dysplasia and is the strongest risk factor for esophageal adenocarcinoma [2, 49]. As the second most common form of esophageal neoplasm after squamous cell carcinoma, esophageal adenocarcinoma has a very poor long-term outcome with a high mortality [3, 50].

Fortunately, several very large longitudinal studies suggest that only a minority of GERD sufferers develop Barrett's esophagus [51, 52]. These studies found that Barrett's esophagus developed in 0.0–1.8% of persons with nonerosive esophagitis and in 1.0–9.9% of persons with erosive esophagitis. Thus, the overall risk of development of Barrett's esophagus in GERD sufferers is low, though generally increasing, with a slightly elevated risk in individuals with erosive esophagitis.

A very low incidence of 1.0 per 100,000 for esophageal adenocarcinoma was reported in male American White and non-Hispanic GERD suffers aged below 50 years, which increased for older men to reach an incidence of 60.8 per 100,000 in 70-year olds [53]. The risk in women was very low across all age groups, increasing to 3.9 per 100,000 at 60 years. Based on these findings, recommendations for endoscopic examinations for adenocarcinoma were not advised in men aged less than 50 years and in women of all age groups, regardless of GERD symptoms [54].

However, a recent systematic review and meta-analysis of population-based studies found associations between frequent GERD symptoms and esophageal adenocarcinoma, with weekly and daily symptoms increasing the odds ratio of esophageal carcinoma by fivefold and sevenfold, respectively [3]. In a population-based case-control study investigating the association between obesity, GERD, and esophageal adenocarcinoma in White Australians, a greater risk of progression of adenocarcinoma was observed in men than in women [55]. The relative risk of adenocarcinoma was alarmingly higher in obese individuals who experienced frequent GERD symptoms than in obese persons with no GERD symptoms. Pooled data on esophageal adenocarcinoma and cigarette smoking showed that smoking also increased the risk of esophageal adenocarcinoma [56]. However, a recent review reported inconsistent associations between diets (containing meat and high-fat levels) and esophageal changes (including Barrett's esophagus, esophageal adenocarcinoma, and esophagogastric junction adenocarcinoma) [57]. These findings provide some information about possible risk factors for Barrett's esophagus and adenocarcinoma, but caution is needed when interpreting the results because of the lack of control over GERD, as a confounding variable. In this context, further research is needed to clarify the roles of lifestyle factors and their interaction with GERD in causing Barrett's esophagus and esophageal adenocarcinoma.

## 7. Extraesophageal Manifestations of GERD

Extraesophageal manifestations possibly resulting from GERD include laryngeal (reflux laryngitis, hoarseness, chronic cough, vocal cord ulcer, and granuloma), pharyngeal (mucositis), respiratory (asthma, bronchitis, chronic cough, and aspiration pneumonia), sinus (sinusitis), middle ear (otitis media), and oral conditions (tooth erosion and sensitivity, sour taste, halitosis, and mucositis) [4, 6, 58, 59]. Oral manifestations of gastric conditions have been largely ignored in the gastroenterology literature, though a recent gastroenterology textbook very briefly included this topic in an attempt to provide a holistic approach for the management of several gastrointestinal conditions [41].

Oral mucosal lesions may result from GERD by direct acid or acidic vapor contact in the oral cavity [41]. However, there is a paucity of information on the effect of GERD on oral mucosal changes. One large case-control study observed a significant association of GERD with erythema of the palatal mucosa and uvula [60]. A histologic examination of palatal mucosa found a greater prevalence of epithelial atrophy, deepening of epithelial crests in connective tissue, and a higher prevalence of fibroblasts in 31 GERD patients compared with 14 control subjects [61]. But these changes were not visible to the naked eye, and the risk of any progression to carcinoma was not known. Though this same study of persons with and without GERD reported a lack of significant differences in salivary flow rates, buffering capacity, and pH values [61], the more recent large case-control study found a significant association between GERD and xerostomia [60].

## 8. Interaction between Endogenous Acid and Saliva

Although the functions of saliva are too many to detail in this paper, it is well established that saliva plays a major protective role in the oral cavity [62]. Apart from providing all the raw ingredients necessary for the repair of hard tooth tissue by remineralization [63], the buffering action of saliva in both the resting and particularly in the stimulated states is one of its most important attributes [62]. These two functions are enhanced by saliva's antibacterial and antifungal properties that inherently control the nature of the oral biofilm acting as a protective entity. It can be argued that these and many other functions are evidence of a “balanced” symbiotic relationship existing between the host and the oral biofilm. A breakdown of this balance often leads to disease.

The functions outlined are often used as evidence for the protective role of saliva against endogenous and exogenous acids. Although this protective role appears logical, it can be argued that saliva has little protective ability in severe erosive conditions. Endogenous acid has a pH of approximately 1.2, which is well below the critical pH for dissolution of hydroxyapatite and fluorapatite [64, 65]. And the acid often acts on tooth structure in situations where the saliva is compromised both in quality and quantity. Even if the saliva is not compromised, such low pH acidic environments cause rapid demineralization of tooth surfaces for a number of reasons.

**Figure 2:**
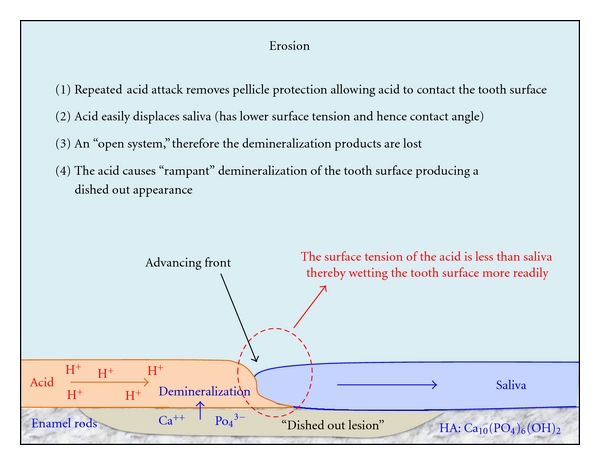
When dental pellicle is removed by sustained endogenous acid attacks, then demineralized tooth products are lost to the oral environment. HA: hydroxyapatite. (Amended Figure  2.5, page 15, from Smales et al. [67]. With copyright permission from Jaypee Medical Publishers 2011.)

The dynamic interaction between different fluids such as various acids and saliva is determined by several factors, the most important being the surface tension of each fluid and the contact angles that each advancing fluid front forms with the tooth surface (Figure 2). As a general rule, acids will displace saliva easily, while saliva will not readily displace acids [66]. There is a strong case that the presence of saliva has no direct benefit or protection against endogenous acid erosion, which may occur initially when only resting saliva is present.

In addition, the surfaces of the teeth during active endogenous acid erosion are largely devoid of protective biofilm and stains due to gastric acid, and also possible proteolytic pepsin. This is an “open system” where the raw products resulting from hard tooth tissue demineralization are lost and are not available to be reused when the oral pH increases back to normal levels [67]. The chemical action causes rapid dissolution of exposed tooth surfaces that is distinctly different from the subsurface dissolution seen with plaque acids [68]. Under magnification, the eroded tooth surfaces will show damage to the ends of the enamel rods, which will only remineralize after the endogenous acid has been cleared from the oral cavity and after salivary pellicle has been reestablished on the tooth surfaces.

The addition of remineralizing ions to the eroded surfaces will only result in the repair of the ends of the enamel rods as the “gross” surface damage is irreversible. Even when fluorapatite is present in high concentrations, the remineralized surfaces provide little or no extra protection to further sustained demineralization as the endogenous acid has a pH well below 4.5, which is the approximate critical pH for fluorapatite dissolution [69]. These findings are supported by observations that fluoride-based and casein-based (amorphous calcium phosphate stabilized by casein-phosphopeptide) remineralizing agents provide some protection against erosion at pH 3.0 [70–73], but not at a highly erosive environment of pH 1.2 [74, 75].

As a result, the principal method for preventing the endogenous tooth erosion from occurring is to eliminate the primary cause, requiring a close relationship with the patient's medical practitioner. The success of medical intervention is quite variable among patients, and their treatment is often difficult to manage. From the dental practitioner's perspective, any possible exogenous dietary and other acids that may be contributing to the problem also need to be eliminated and saliva production stimulated.

In addition to attempting to eliminate the primary cause, the placement of any physical barrier between the tooth surfaces and the endogenous acid should be of benefit. Many “metal ion” fluorides such as SnF, AgF, TiF_4_, and FeF_3_ have been tested and do show some laboratory evidence of a protective effect [76]. The mechanism of action is probably not by the fluoride ion itself, but by the metal ion precipitate that forms a physical barrier to the acid. Other dental products that can be used as, often, temporary physical barriers to acid include resin-based viscous varnishes, resin-based dentin bonding agents [77], and a thin layer of an unfilled/lightly filled clear adhesive resin sealant or glass-ionomer cement [78]. Alternatively, casein-based remineralizing paste acts as an artificial biofilm that contains all the raw products for tooth tissue remineralization [79]. However, surface barrier products generally require testing in independent controlled trials to identify their efficacy and long-term clinical cost effectiveness.

## 9. Association between Tooth Erosion and GERD

**Figure 3:**
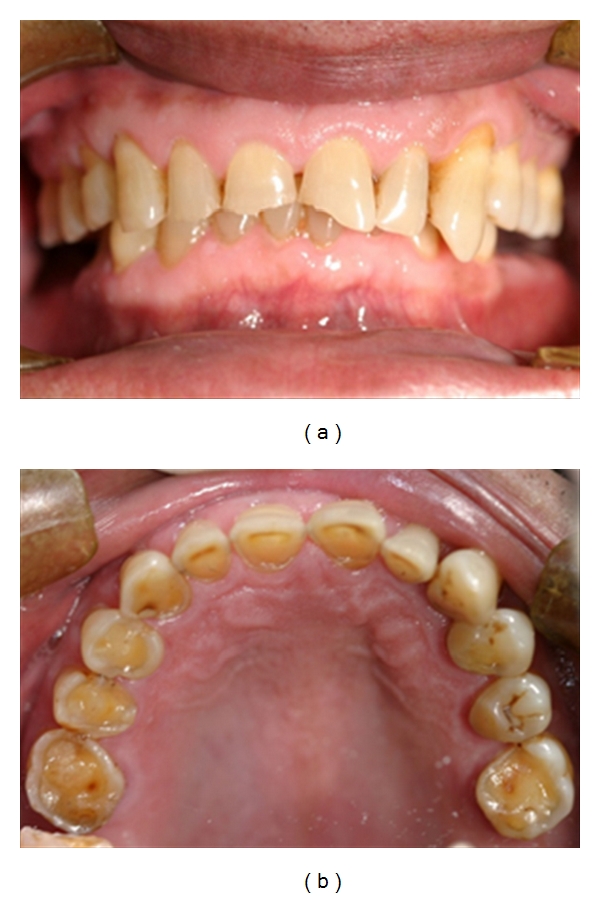
Frontal and maxillary occlusal views of severe tooth erosion caused by endogenous acid in a patient with GERD. (Courtesy of Dr. A. Dickson.)

Dental erosion or, more correctly, corrosion is described as tooth surface loss produced by chemical or electrolytic processes of nonbacterial origin, which usually involves acids [80]. The acids are of endogenous (intrinsic) origin from refluxed gastric juices (Figure 3) and/or of exogenous (extrinsic) origin from usually dietary, medicinal, occupational, and recreational sources. The erosive potential of the acids is modified by many secondary factors.

As part of what is known as the Montreal consensus, 44 physicians from 18 countries voted on the statement that “The prevalence of dental erosions, especially on the lingual and palatal tooth surfaces, is increased in patients with GERD” [17]. Although the result was a high-grade consensus agreement of 96%, only 42% of the votes “agreed strongly” with the statement, 35% “agreed with minor reservations,” and 19% “agreed with major reservations.” Only three selected clinical studies quoted to support the statement [81–83]. The global consensus report also stated that extraesophageal syndromes rarely occurred in isolation without concomitant manifestations of the typical esophageal syndrome, that these association syndromes are usually multifactorial with GERD as one of several potential aggravating cofactors, and that data substantiating a beneficial effect of reflux treatments on the extraesophageal syndromes are weak [17].

 Subsequently, eight pediatric gastroenterologists using a revision of the original Montreal protocol voted on the statement that “GERD may cause dental erosions in pediatric patients” [18]. The result was a low-grade consensus agreement of 100%, with just 12.5% of the votes “agreed strongly,” 37.5% “agreed moderately,” and 50% “just agreed.” One systematic review article [7] and four other selected clinical articles [84–87] were quoted to support the statement. Dental erosion was only one of two extraesophageal syndromes considered to be definitely associated with GERD in pediatric patients, the other being Sandifer's syndrome (torticollis) [18]. Asthma and laryngopharyngeal syndromes were not considered to be definitely associated with GERD in children, unlike their established associations in adults [17]. The reporting of symptoms caused by GERD is likely to be unreliable in children under the age of approximately eight years and in older persons lacking sufficient cognitive abilities.

Tooth erosion associated with GERD was apparently first reported in 1933 [88]. However, apart from the subsequent occasional publications of case reports, until the 1990s few research publications evaluated this association. Several of these later research studies were discussed by Bartlett [4] and by Wong et al. [8] who concluded, respectively, that there was a clear though variable relationship between GERD and dental erosion and that controlled clinical studies were required to show that the progression of dental erosion ceased after adequate gastric acid suppression therapy in patients with GERD.

 A recent systematic review involving 17 eligible mainly observational and case-control studies of GERD and dental erosion found a strong association between the two conditions [7]. The median prevalence of dental erosion in GERD patients was 24%, and the median prevalence of GERD in adults and in children with dental erosions was 32.5% and 17%, respectively. However, there were wide percentage ranges and degrees of tooth tissue loss present among the study populations, and not all studies and evaluations of patients employed esophageal endoscopy and/or 24-h esophageal pH-metry. One other recent systemic review also found a higher prevalence of dental erosion, asthma, pneumonia, and sinusitis in children with GERD compared with healthy controls [5]. The authors could not find any eligible studies in children with GERD that investigated associations with bronchitis, cough, laryngitis, pharyngitis, and sleep apnea.

 A recent study of 249 referred children and adult Icelandic persons, of whom 91 had molar erosion and/or symptoms of gastric reflux and had undergone gastroscopy, esophageal manometry, and 24-h esophageal pH-metry, found a significant association between diagnosed GERD and dental erosion [48]. The severity of dental erosion in the incisor and the molar teeth was assessed separately using modified criteria from an erosion index [89]. Step-wise logistic regression analyses showed significant associations in particular between diagnosed regurgitation and palatal erosion, the daily consumption of more than 0.5 L of acidic drinks, and a low buffer capacity of saliva. However, combining all of the factors measured in the study only provided a low explanatory value of 15.1% for the variability in erosion scores.

 By contrast, a large case-control study of men and women aged from 19 to 78 years found no significant associations between GERD and either dental erosion or tooth sensitivity, but significant associations between GERD and xerostomia, oral acid/burning sensation, subjective halitosis, and erythema of the palatal mucosa and uvula [60]. GERD was diagnosed in all new patients seen at the Gastroenterology Department using esophagogastroduodenoscopy and 24-h esophageal pH-metry. And dental erosion was evaluated using a Tooth Wear Index [90], which is not restricted to assessing tooth tissue loss from erosion alone. However, only 9% of the 200 patients with GERD and 13% of the 100 healthy matched controls showed evidence of any dental erosion (tooth tissue loss). Both groups of subjects had similar tooth sensitivities of 32.5% and 32%. It was postulated that a significant portion of cases (with dental erosion) reported in the literature could have consisted of patients with a particularly abundant reflux or who were unresponsive to pharmacological therapy [60].

 The first randomized clinical trial to demonstrate quantitatively the short-term suppression of active tooth erosion following the treatment of medically confirmed GERD with a proton pump inhibitor (PPI) has recently been published [91]. Subjects with other causes for dental erosion, and who failed to meet additional selection criteria, were excluded from the study. Optical coherence tomography was used to quantify the extent of enamel demineralization at multiple specific sites in specific visibly eroded teeth both before and after three weeks of esomeprazole therapy. In this double-blinded study, there was significantly less enamel thickness lost in the 14 available adult subjects taking esomeprazole (mean = 7.20 *μ*m) than in the 15 adult subjects taking a placebo (mean = 15.25 *μ*m). Evidence of a mild remineralization of eroded teeth in the esomeprazole subjects was shown by a decreased optical reflectance at a depth of 25 *μ*m. Because nocturnal acid control may be inadequate with PPIs, some erosion from GERD may have continued during sleep. Most of the patients had mildly symptomatic GERD, as they presented with a primary complaint of dental erosion.

 Studies that attempt to associate tooth erosion with the findings from esophageal pH-metry often only assess gastric reflux occurring classically 5 cm above the LES. However, the refluxate will only enter the oropharynx once the upper esophageal sphincter (UES) has been breached. A significant correlation of palatal tooth erosion with acid reflux was demonstrated in a study of 31 adult patients that employed 24-h esophageal pH-metry with dual electrodes located 5 cm above the LES and 2 cm above the UES [92]. There were significant correlations between the proportion of the total time (and also of the supine time) that pharyngeal pH was below 5.5, and the proportion of teeth with obvious palatal wear scores. (The critical pH for enamel demineralization is approximately 5.5.) The authors concluded that the pH (below 4.0) criterion accepted for the diagnosis of GERD at 5 cm above the LES was probably not relevant to the pharynx.

 Using male Wistar rats, an animal model was developed to determine the effects of forced and continuously occurring gastroduodenal reflux, following esophagojejunostomy without gastrectomy, on tooth erosion [93]. After 30 weeks, the pH of the gastric contents in the forced reflux and sham-operated control rats was 3.70 and 3.36, respectively. At this time the pH of the esophageal contents in the sacrificed reflux rats was 6.93 and was associated with extensive tooth erosion in the molar teeth. (Almost no tooth erosion was observed in the sham-operated rats.) The refluxate was a mixture of saliva, gastric, and duodenal contents that included bile secretions and probably also acidic vapor. In humans, high intraesophageal refluxates have been shown to contain a mixed liquid-gas composition and to be significantly associated with GERD symptoms irrespective of an LES recorded pH above or below 4.0 [94]. Endogenous tooth erosion in the absence of GERD symptoms (silent refluxers) could be caused by acidic droplets/vapors and gases.

 When compared with a control group of healthy subjects, an increased prevalence of tooth erosions was significantly associated with an increased frequency of respiratory symptoms in a recent clinical study of 88 carefully selected adult patients with medically confirmed GERD [95]. Palatal erosion of maxillary incisors was found in 80% of patients with frequent respiratory symptoms such as chronic cough, laryngitis, and asthma. Strong associations have been reported between GERD and asthma [17] and between asthma and tooth erosion [96]. Some of these associations are linked to the systemic effects of ingested and inhaled drugs in decreasing the saliva flow and LES tonus and to the acidic nature of powdered topical drugs contained in puffers that are used to treat asthma.

## 10. Conclusions

GERD is an increasingly common and potentially serious condition, with various extraesophageal adverse health effects that dental practitioners should be aware of. Clinicians should also be aware of the predisposing risk factors for GERD and its classical esophageal and extraesophageal symptoms and signs. However, not all affected persons will have the classical symptoms of gastric regurgitation. Dentists may be the first persons to diagnose the possibility of GERD in these “silent refluxers,” particularly when observing unexplained instances of tooth erosion, which might be accompanied by coexisting hyposalivation. Numerous laboratory, and mainly case-control and observational clinical studies in adults and children, have shown a clear though variable relationship between GERD and tooth erosion. However, further randomized clinical trials are required to demonstrate that the progression of dental erosion reduces or ceases following gastric acid suppression therapy in patients with confirmed GERD. Collaborative medical and dental management of patients with GERD is strongly advocated.
